# Strategies to Obtain Encapsulation and Controlled Release of Small Hydrophilic Molecules

**DOI:** 10.3389/fbioe.2020.00437

**Published:** 2020-05-13

**Authors:** Qi Li, Xiaosi Li, Chao Zhao

**Affiliations:** Department of Chemical and Biological Engineering, The University of Alabama, Tuscaloosa, AL, United States

**Keywords:** small, hydrophilic, drug, encapsulation, controlled release

## Abstract

The therapeutic effect of small hydrophilic molecules is limited by the rapid clearance from the systemic circulation or a local site of administration. The unsuitable pharmacokinetics and biodistribution can be improved by encapsulating them in drug delivery systems. However, the high-water solubility, very hydrophilic nature, and low molecular weight make it difficult to encapsulate small hydrophilic molecules in many drug delivery systems. In this mini-review, we highlight three strategies to efficiently encapsulate small hydrophilic molecules and achieve controlled release: physical encapsulation in micro/nanocapsules, physical adsorption via electronic interactions, and covalent conjugation. The principles, advantages, and disadvantages of each strategy are discussed. This review paper could be a guide for scientists, engineers, and medical doctors who want to improve the therapeutic efficacy of small hydrophilic drugs.

## Introduction

**FIGURE 1:**
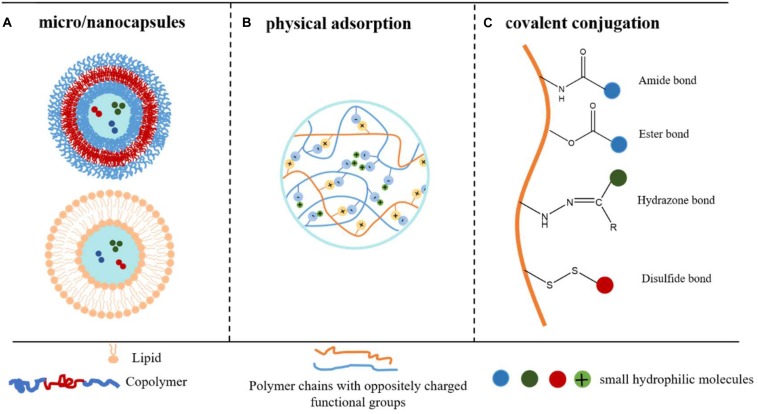
Strategies to obtain encapsulation and controlled release of small hydrophilic molecules. **(A)** Drugs are physically encapsulated inside the aqueous pockets of the micro/nanocapsules. **(B)** Drugs are physically encapsulated in the polymer particles via electronic interaction. **(C)** Drugs are chemically conjugated onto polymer backbones via covalent bonds.

Small hydrophilic molecules are widely used for treating diseases such as infectious diseases (Macielag, 2012; Zhang et al., 2015; Chandel et al., 2018), cancer (Xu et al., 2014; Zhao et al., 2016), and local anesthesia (Howell and Chauhan, 2009; Jug et al., 2010). Although effective, the dosage, therapeutic effect, and patient accomplishment of such compounds are usually limited by the tendency to distribute into the biological aqueous environment of the human body, leading to side effects (Weiniger et al., 2010; Wang et al., 2019). The pharmacokinetics and biodistribution profile of small hydrophilic molecules can be improved by encapsulating them in delivery systems which allow the sustained release and prolonging retention period. However, due to the good water solubility, hydrophilic nature, and low molecular weight, such compounds have weak interactions with many conventional drug carriers, such as hydrogels (Yu et al., 2013), poly(lactic-co-glycolic acid) microspheres (Ramazani et al., 2016), and electrospinning fibrous mat (Oliveira et al., 2015; Sultanova et al., 2016), leading to low encapsulation efficiency, undesired leakage, and initial burst release. Although many delivery systems have been attempted and shown promise in encapsulation and sustained release of hydrophilic molecules (Vrignaud et al., 2011), most of them only work well for molecules with the moderate hydrophilicity and medium molecular weight. When it comes to the super hydrophilic and very small molecules, their effectiveness is not adequate. In this review, the emphasis was given to the group of super challenging small hydrophilic molecules: compounds that have a molecular weight below 1000 Da and have a logP (partition coefficient, or XLogP3, a computed form of logP) or logD (distribution coefficient) value less than 3.0 under their administration condition. In particular, tetrodotoxin (TTX, Mw 319.27 g/mol, LogP = −1.89), one of the most challenging compounds to encapsulate because it is small and very hydrophilic, was selected as a reference. We introduce three efficient strategies that have been validated to encapsulate TTX and to achieve sustained TTX release, including physical encapsulation in micro/nanocapsules, physical adsorption via electronic interactions, and covalent conjugation (Figure 1). The advantages and limitations of each strategy were summarized (Table 1).

**TABLE 1:** Advantages and limitations of strategies for small hydrophilic molecules.

	Advantages	Limitations
Micro/nanocapsules	✓ High drug encapsulation efficiency ✓ Good biocompatibility ✓ High modifiability	✓ Instability in plasma ✓ Leakage during storage ✓ High cost ✓ Toxicity related to solvents
Physical adsorption	✓ Easy operation ✓ High biocompatibility ✓ Less toxicity related to solvents and chemical crosslinking agents	✓ Initial rapid drug release ✓ Less controllability
Covalent conjugation	✓ Controllable drug loading ✓ Enhanced stability ✓ Stimuli-responsive release	✓ Toxicity related to solvents and coupling agents

## Physical Encapsulation in Micro/Nanocapsules

Micro/nanocapsules are colloidal drug carrier systems composed of aqueous pockets surrounded by a hydrophobic membrane (Couvreur et al., 2002). Based on whether the shell is composed of lipids or polymers, the capsules are categorized as “liposomes” and “polymersomes,” respectively. Liposomes (Torchilin, 2005; Chen, 2010; Eloy et al., 2014) and polymersomes (Levine et al., 2008; Anajafi and Mallik, 2015; Müller and Landfester, 2015) have been properly summarized in many other review articles. Here, we only briefly highlight the principles of encapsulating drugs in them and their associated advantages and limitations.

Liposomes and polymersomes encapsulate small hydrophilic molecules inside the internal aqueous pockets to achieve a high encapsulation efficiency. The hydrophobic shell prevents the encapsulated drug from rapid clearance, achieving sustained release (Figure 1A).

There are three types of liposomes: multilamellar vesicles, small unilamellar vesicles, and large unilamellar vesicles. The encapsulation efficiency is highly influenced by the liposome size and the drug release rate is determined by the liposome stability and shell permeability (Taylor et al., 1990; Glavas-Dodov et al., 2005). A larger internal volume leads to the higher efficiency of drug loading, while a stable liposome structure avoids the leakage of small molecular hydrophilic drugs. These essential parameters of liposomes can be adjusted to a great extent by the lipid membrane composition, chain length of the phospholipid, drug to lipid ratio, and charge property (Eloy et al., 2014).

Many liposomal formulas for small hydrophilic drugs have been FDA approved and marketed due to the high drug encapsulation efficiency, extended drug half-time, and excellent biocompatibility (Fan and Zhang, 2013). For example, DOXIL^®^, Myocet^®^, and CAELYX^®^ are marketed liposomal formulations for doxorubicin hydrochloride (DOX-HCl, Mw 580 g/mol, logD = −0.45 at pH 5.8; Dubbelboer et al., 2014), and the DAUNOXOME^®^ is a marketed liposomal formulation for daunorubicin (Mw 527.5 g/mol, LogP = 1.83) (Chen, 2010).

**FIGURE 2:**
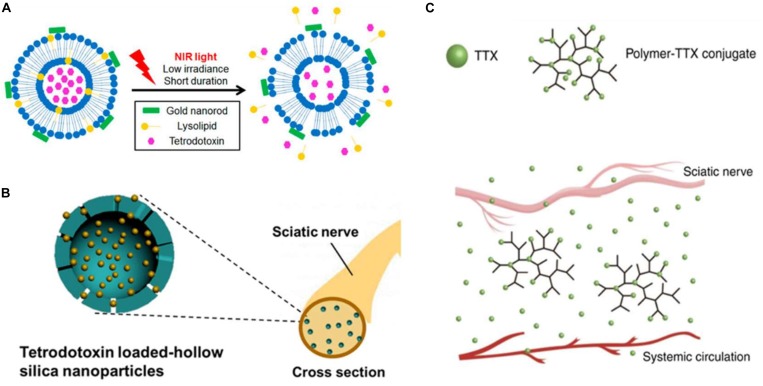
Strategies that have been validated to encapsulate tetrodotoxin (TTX). TTX is a naturally occurring potent sodium channel blocker. Being small and super hydrophilic, TTX is selected as the gold standard for the drug delivery systems in this review. **(A)** Schematic of the photo-triggerable liposomes loaded with TTX (reprinted with permission from *Nano Lett. 2017, 17, 2, 660–665*). **(B)** Schematic of TTX-loaded hollow silica nanoparticles (reprinted with permission from *Nano Lett. 2018, 18, 1, 32–37*). **(C)** Schematic of the polymer-TTX conjugate, a (reprinted with permission from *Nat Commun. 2019, 10, 2566*).

Kohane and colleagues encapsulated TTX into liposomes and functionalized the liposome shell with gold nanorods (Zhan et al., 2016; Guo et al., 2020), photosensitizer (Rwei et al., 2015, 2017b), and sonosensitizer (Rwei et al., 2017a; Cullion et al., 2018), making the liposome sensitive to effects of near-infrared (NIR) light and ultrasound. The prepared formulations could release the encapsulated TTX to treat pain after operations with on-demand irradiation (Figure 2A).

The applications of liposomes are still limited by (1) instability in plasma: untailored or unmodified capsules can be adsorbed by human albumin or serum and further cleared by the immune system, inducing a short half-life in blood circulation (Wakaskar, 2018); (2) leakage during storage: the permeability of the lipid bilayer could cause leakage of the capsuled molecules during the formation process and the afterward storage (Zariwala et al., 2018), inducing an initial burst release when implemented after storage; and (3) high cost due to the expensive raw lipid materials and cumbersome production procedures.

Polymersomes have many advantages over liposomes, which give them greater potential as drug carriers. They are more stable and less permeable than liposomes due to their membrane thickness, entanglement, and lateral diffusivity (Chandrawati and Caruso, 2012; Rideau et al., 2018). Properties of polymersomes, including size, permeability, and charge property, are far more versatile than that of liposomes due to the abundantly available natural and synthetic polymer (Anajafi and Mallik, 2015; Dan, 2018; Rideau et al., 2018). The advances of polymer chemistry allow the conjugation of active ligands, functional molecules, antibodies onto the polymer, enabling polymersomes the functions of targeted and stimuli-responsive (pH, redox, enzyme, ultrasound, magnetic field, light) drug delivery (Levine et al., 2008; Müller and Landfester, 2015; Leong et al., 2018).

However, the clinical applications of polymersomes are hampered by the residual organic solvent, incompetent control of the early drug release, cumbersome fabrication steps, and toxicity concerns (Vrignaud et al., 2011; Anajafi and Mallik, 2015).

## Physical Adsorption

Physical adsorption strategy refers to that the molecules are physically adsorbed on carriers via inter-molecule interactions, such as ionic interaction, H-bond, van der Waals forces, hydrophobic interactions, dipole–dipole interactions (Margenau and Kestner, 2013). For the delivery of small hydrophilic molecules, ionic interaction is more preferred since others are weaker and less efficient (Yu et al., 2017) (Figure 1B).

Such a strategy is commonly utilized to load drugs into nanoparticles, silica nanoparticles, magnetic nanoparticles (Guo et al., 2020), and carbon nanodots (Tang et al., 2013). Yu utilized bioactive glass nanoparticles (BGNs) as carriers to load two different model drugs—diclofenac sodium (DS, Mw 318.13 g/mol, LogD = 1.1 at pH 7.4) and 5-fluorouracil (5-Fu, Mw 130.08 g/mol, XLogP3 = −0.9) (Carrer et al., 2018). GNs demonstrated ∼45 folds improvement of DS loading because of the strong ionic interaction between the calcium iron of BGNs and the carboxylate group of the drug. On the contrary, BGNs showed limited loading capability to 5-Fu because there are weak electronic interactions between BGNs and 5-Fu (Yu et al., 2017). Liu loaded the positively charged TTX into the negatively charged silica nanoparticles through electronic interactions (Figure 2B). The resulted formulation achieved sustained TTX release and decreased TTX toxicity (Liu et al., 2018).

Polyelectrolytes (PEs) are polymers that have repeating units bearing electrolyte groups. When placed in the ionizing solvent, PE will dissociate into polycations and polyanions. Then, ionized PEs in the solution can form a complex with oppositely charged PEs—a PE complex (PEC) (Meka et al., 2017). Such “chaotic” aggregation of polyanions and polycations might only be the result of partial mutual charge compensation, leaving a huge number of ionic sites compensated by small molecules with counter ions to preserve the electro neutrality (Philipp et al., 1989).

One major type of PE is natural polysaccharide such as chitosan, auricularia auricular polysaccharide (Xiong et al., 2016), alginate, and hyaluronic acid. They are charged due to the possession of a considerable number of charged functional groups such as carboxyl and amino groups (Liu et al., 2008), which cannot only capture and entrap hydrophilic drugs but also compact the polymer chains into stable nanoparticles.

The physical adsorption method can be considered as an energy-efficient way to achieve a high loading capacity by merely mixing the small molecules and carriers under ambient temperature. Physical adsorption is also advantageous in the diversity of polymers, which allowed the regulation of drug encapsulation efficiency, drug release profile, physical/chemical property, and biocompatibility of the PEC (Philipp et al., 1989). Besides, this strategy reduces the use of solvents and chemical crosslinking agents, addressing the potential toxicity problem (Lankalapalli and Kolapalli, 2009).

One shortcoming associated with the physical adsorption strategy is the initial rapid drug release, which is due to the saturation of the counter-ions of the carriers or the fast ion exchange (Xiong et al., 2016; Yu et al., 2017). Besides, the release profile of the drug significantly impacts on the pH, the salt concentration of the environment, which makes the drug release less controllable (Kulkarni et al., 2016).

## Conjugate Delivery System

In the conjugate delivery system, small hydrophilic molecules are covalently bonded onto the polymer or lipid chains through cleavable linkage, turned to be prodrugs and applied in a wider range of release routes (Figure 1C) (Adhikari et al., 2017; Irby et al., 2018; Markovic et al., 2019).

Conjugate delivery systems overcome the main drawbacks of non-covalent physical methods, unfavorable leakage and burst release, due to stable linkers between drugs and polymers (Chen et al., 2014). Zhao et al. (2019) synthesized a range of rationally designed PEGylated and non-PEGylated polymers to which the ultra-potent local anesthetic TTX was conjugated by hydrolysable ester bonds. Zhao demonstrated that TTX was released in its native form, and the release rate can be regulated by manipulating the polymer composition (the TTX release rate is proportional to the hydrophilicity of polymer backbone). *In vivo*, the polymer-TTX conjugate obviated TTX burst release to allow the administration of 80 μg of TTX into rats, which that is 20-fold higher than the reported dose tolerance limits. The described formulation produced a sciatic nerve blockade lasting for 3 days in rats but did not cause any animal death or adverse effects (Figure 2C).

Conjugated delivery systems allow drug loading to be controlled by adjusting the drug-to-polymer ratio (Singh et al., 2008). The amount of drug loaded depends on the number of reactive sites on the backbones. Besides, through selecting covalent bonds, stimuli-responsive drug release can be achieved (Dutta et al., 2019). For example, hydrazone bonds show strong stability under a neutral pH environment and sustained release in a lower pH environment (Tang et al., 2018). The disulfide bond is widely used as a reduction-responsive linker (Sun et al., 2018). These bonds can facilitate rapid and differential release of chemotherapeutic drugs in tumor cells to achieve the tumor-targeted drug delivery (Chang et al., 2019).

Through chemical bonds, some inapplicable delivery system for small hydrophilic molecules can be preferable. Micelles have the typical structure containing a hydrophilic shell and a hydrophobic core. Based on the structure, micelles were investigated for encapsulating hydrophobic drugs because of the hydrophobicity of the inner core (Li et al., 2011; Jhaveri and Torchilin, 2014). While the hydrophobicity decreases their interactions with the hydrophilic drugs, leading to low loading capacity. The high and stable hydrophilic drug loading in micelles could be achieved by covalently linking the drug with polymer backbone (Liao et al., 2016; Danafar et al., 2017).

Coupling agents, catalysts, and solvents are used for the covalent conjugation of drug to polymers. The compound residue usually causes concern over their toxicity.

## Conclusion and Perspectives

Physical encapsulation in micro/nanocapsules, physical adsorption via electronic interactions, and covalent conjugation are the most efficient strategies to improve the therapeutic efficacy and to minimize side effects of small hydrophilic drugs. Among them, liposomal formulations have been clinically used due to the excellent lipid biocompatibility. The biocompatibility of other materials needs to be carefully examined before their clinical practice. Each strategy has its advantages and limitations. The selection of a delivery method depends on the drug property, desired drug dose, and the preferred drug release profile.

The route of administration would affect the effectiveness of the strategies in encapsulating small hydrophilic drugs. The three strategies described in this review would show good controlled release for the drugs administrated by the intramuscular, subcutaneous, intradermal injections. However, their effectiveness may be significantly reduced for oral and intravenous administrated drugs. The enzymatic digestion at acidic pH in the stomach would rapidly destroy the carrier structure and/or drug–carrier interaction, leading to the burst drug release. The long-term circulation in the blood would lead to the drug’s early release before the carriers reaching to the target sites. The encapsulation of oral and intravenous administrated small hydrophilic molecules into carriers that could considerably improve the drug efficiency would be a significant need in the future.

## Author Contributions

QL, XL, and CZ wrote the manuscript.

## Conflict of Interest

The authors declare that the research was conducted in the absence of any commercial or financial relationships that could be construed as a potential conflict of interest.
